# Absolute eosinophil count may be an optimal peripheral blood marker to identify the risk of immune-related adverse events in advanced malignant tumors treated with PD-1/PD-L1 inhibitors: a retrospective analysis

**DOI:** 10.1186/s12957-022-02695-y

**Published:** 2022-07-28

**Authors:** Yan Ma, Xiao Ma, Jingting Wang, Shanshan Wu, Jing Wang, Bangwei Cao

**Affiliations:** 1 Present Address: Shijingshan Teaching Hospital of Capital Medical University, Beijing Shijingshan Hospital, #24 Shi Jing Shan Road, Beijing, Shijingshan District 100040 China; 2 Department of Oncology, Beijing Friendship Hospital, Capital Medical University, #95 Yong An Road, Beijing, 100050 Xicheng District China; 3 Department of Clinical Epidemiology and EBM, Beijing Friendship Hospital, Capital Medical University, Beijing, 100050 China

**Keywords:** Immunotherapy, Immune-related adverse events, Neutrophil-to-lymphocyte ratio, Platelet-to-lymphocyte ratio, Absolute eosinophil count, Predictive markers

## Abstract

**Background:**

This study aimed to investigate the predictive values of serum biomarkers including absolute eosinophil count (AEC), neutrophil-to-lymphocyte ratio (NLR), and platelet-to-lymphocyte ratio (PLR) with respect to immune-related adverse events (irAEs) during anti-PD-1/PD-L1 inhibitor treatment in patients with advanced malignant tumors.

**Methods:**

We retrospectively analyzed 95 patients with advanced cancer who were treated with anti-PD-1/PD-L1 inhibitors from January 1, 2017, to May 1, 2020, in our cancer center. We then analyzed associations between irAEs and anti-PD-1/PD-L1 inhibitor responses and evaluated the predictive values of serum biomarkers with respect to the risk of irAEs.

**Results:**

The incidence of irAEs was 55.8%. There were no statistically significant differences between the irAEs and no-irAEs groups in an objective response rate (ORR) or disease control rate (DCR). However, landmark analysis showed that the irAEs group had better survival after 120 days following the initiation of anti-PD-1/PD-L1 inhibitor treatment, compared with the no-irAEs group. The incidences of irAEs were greater in the high-AEC and low-NLR groups than in the low-AEC and high-NLR groups. Univariate logistic analysis showed that low NLR, ECOG performance status (0–1), and high AEC were risk factors for irAEs. Multivariate logistic analysis showed that high AEC and good ECOG performance status were independent predictors for irAEs.

**Conclusions:**

irAEs may be associated with a survival benefit. Baseline AEC is a strong predictor of irAEs in patients undergoing treatment with anti-PD-1/PD-L1 inhibitors.

## Introduction

Immune checkpoint inhibitor therapy, represented by PD-1/PD-L1, has been widely used for the treatment of many advanced malignant tumors, with significant and sustained efficacy; it has had a robust impact on traditional treatment modalities such as cytotoxic chemotherapy and targeted therapy [1–5]. Despite its favorable effect, the immune-related adverse reactions that occur during immune checkpoint inhibitor treatment should not be ignored.

irAEs are broadly defined as instances of immune-mediated host organ dysfunction caused by abnormal immune system activity following immunotherapy [6]. In brief, immune checkpoint inhibitors activate the immune system, enhance the immune response, cause a large number of inflammatory cytokines to attack normal organs, and produce a variety of toxic side effects. Despite extensive investigation, the underlying mechanisms of irAEs remain unclear. Generally, treatment with PD-1/PD-L1 inhibitors activates transcription factors that alter the epigenome of depleted T cells, ultimately causing the immune system to reactivate and attack “self” tissue [7].

irAEs reportedly may occur in up to three-quarters of patients who undergo treatment with combined immune checkpoint inhibitors [8]. They are most common in the skin, thyroid, and gastrointestinal tract, although they may involve any organ or system, including the heart, lungs, liver, and pituitary gland [9]. Immune-related adverse events (irAEs) are usually easy to manage; however, approximately 10% of affected patients experience irAEs sufficiently severe to require discontinuation of immune checkpoint inhibitor therapy and/or additional treatment with hormonal or immunosuppressive agents [10, 11]. In some instances, irAEs can lead to permanent illness, with potential fatality in approximately 1% of affected patients [12]. It is important to note that irAEs can occur at any point in time, including months after treatment withdrawal [13].

Considering these characteristics of irAEs, their diagnosis and prediction are particularly challenging. Early identification of irAEs related to immune checkpoint inhibitor therapy can reduce both morbidity and mortality. Therefore, biomarkers that can predict irAEs at an early stage are urgently needed [14]. Peripheral blood markers such as absolute eosinophil count (AEC), neutrophil-to-lymphocyte ratio (NLR), and platelet-to-lymphocyte ratio (PLR) have attracted substantial attention because of their non-invasive, rapid, stable, and inexpensive characteristics. NLR and PLR can reportedly predict irAE occurrence related to anti-PD-1/PD-L1 inhibitor therapy in patients with non-small cell lung cancer [15, 16]. An increased NLR has been associated with an increased risk of grades 3–4 pulmonary and gastrointestinal irAEs in melanoma patients treated with nivolumab [17]. Moreover, eosinophils in the peripheral blood were also associated with irAEs [18]. Importantly, eosinophils can serve as regulatory or effector cells for antigen presentation. However, eosinophils eventually mediate inflammation by enhancing activated T cell infiltration [19–21]. In several types of advanced cancer, immune checkpoint inhibitor treatment has been associated with elevated eosinophil counts, better clinical responses, and longer overall survival [22–29]. Increased eosinophil counts at baseline and 1 month were associated with an increased overall risk of irAEs grade 2 and above [14]. Baseline characteristics of high AEC (0.125 × 10^9^/L) were associated with an increased risk of immune-associated pneumonia, as well as better clinical outcomes in patients with non-small cell lung cancer (NSCLC) who were receiving immune checkpoint inhibitor treatment [30]. Additionally, baseline absolute eosinophil count was positively associated with the occurrence of endocrine irAEs [31].

Thus far, the relationships between irAEs and anti-PD-1/PD-L1 inhibitor treatment responses remain controversial. irAEs were positively correlated with anti-PD-1/PD-L1 inhibitor efficacy in patients with NSCLC and patients with melanoma [32–36], but there has been some speculation that this correlation is not robust [37, 38]; a negative correlation has been suggested in patients with small-cell lung cancer [39]. Recently, a study by Rogado et al. involving multiple tumor species showed that irAEs were directly associated with favorable objective response rates (ORRs) and progression-free survival in patients receiving anti-PD-1/PD-L1 inhibitors [40].

This study was performed to evaluate the relationships of irAEs with the clinical efficacy of anti-PD-1/PD-L1 inhibitors in the treatment of advanced malignant tumors. It also screened predictors of irAE risk by investigation of peripheral blood biomarkers (e.g., baseline AEC and baseline NLR).

## Patients and methods

### Study design and patient population

This retrospective study included patients with malignant tumors who were admitted to the Cancer Center of Beijing Friendship Hospital affiliated with Capital Medical University from January 1, 2017, to May 1, 2020. All patients had generally complete case data that allowed assessment of efficacy, disease progression or treatment failure, and irAEs. All tumors were pathologically confirmed. The follow-up period began at the initiation of anti-PD-1/PD-L1 inhibitor therapy and ended at the time of disease progression, confirmed death, or August 31, 2020. The following exclusion criteria were used: previous medical history or test results indicating the presence of a definite hereditary disease; autoimmune disease or other serious medical conditions, such as cardiovascular disease (e.g., atrioventricular block, atrial fibrillation, or congestive heart failure) or kidney disease (e.g., hemodialysis); failure to evaluate serological indicators; and absence of serological test results recorded before and after treatment. Thus, this study included 95 patients. Anti-PD-1/PD-L1 inhibitors used for treatment in this study mainly included nivolumab, atezolizumab, sintilimab, and camrelizumab. Immunotherapy-combined treatment was administered concurrently with either targeted therapy or chemotherapy.

The study protocol was approved by Beijing Friendship Hospital’s Institutional Review Board (2020-P2-176-01) and performed in accordance with the tenets of the Declaration of Helsinki.

### Data collection

Characteristics and clinical data of all 95 patients treated with anti-PD-1/PD-L1 inhibitors were recorded, including age, sex, Eastern Cooperative Oncology Group performance status (ECOG PS), tumor type, cancer TNM staging, treatment lines, treatment, clinical efficacy, and PFS. The tumor stage was determined in accordance with the eighth edition of the Union for International Cancer Control (UICC) TNM classification of Malignant Tumors.

CT scans were performed at baseline, as well as after one and two cycles of anti-PD-1/PD-L1 inhibitor treatment or when clinical disease progression was evident. Response to anti-PD-1/PD-L1 therapy was determined using the Response Evaluation Criteria In Solid Tumors (RECIST) criteria, version 1.1. Efficacy was assessed as complete response (CR), partial response (PR), stable disease (SD), and progressive disease (PD). CR and PR refer to ORR; CR, PR, and SD refer to disease control rate (DCR). PFS was recorded from the beginning of treatment until the observation of disease progression or death from any cause.

irAEs were defined as adverse events with a potential immunologic basis that required close monitoring and/or potential intervention with hormonal or immunosuppressive therapies [20]. irAEs were recorded by analysis of medical records, as well as follow-up interviews involving patients and attending physicians. Baseline measurements were defined as the measurements taken within 3 days prior to receipt of anti-PD-1/PD-L1 inhibitor treatment. Baseline peripheral blood data included absolute neutrophil count, absolute lymphocyte count, platelet count, and absolute eosinophil count.

### Statistical analysis

All data were statistically analyzed using SPSS Statistics, version 25.0. Forest plots were drawn with R software, version 4.0.2. Receiver operating characteristic (ROC) curve analysis was used to determine the optimal cutoff values for peripheral blood markers. The chi-squared test was in 2 × 2 tables. Survival curves were estimated by the Kaplan–Meier method, then analyzed using the log-rank test. Landmark analysis was adopted because of the immortal time bias for irAEs. Associations of baseline AEC, NLR, and PLR with irAEs were evaluated by univariate and multivariate logistic regression analysis. *P* < 0.05 was considered statistically significant for all analyses.

## Results

### Patient characteristics

All 95 patients received anti-PD-1/PD-L1 inhibitor treatment. The patient characteristics are summarized in Table 1. The median age was 62 years (range, 30–80 years); most patients (64.2%) exhibited an ECOG PS of 1. First-line and second-line treatments with anti-PD-1/PD-L1 therapy were recorded in 65% of patients. The median PFS was 108 days. There were zero cases of CR (0%), 12 cases of PR (12.6%), 49 cases of SD (51.6%), and 34 cases of PD (35.8%). The ORR and DCR were 12.6% and 64.2%, respectively.

**Table 1:** Patient characteristics (*n* = 95)

Patient characteristics	Patients treated with anti-PD-1/PD-L1 therapy (***n*** = 95), ***n*** (%)
**Age at initiation of anti-PD-1/PD-L1 therapy (years)**	
Median	62
Range	30–80
**Sex**	
Male	66 (69.5)
Female	29 (30.5)
**ECOG**	
0	25 (26.3)
1	61 (64.2)
2	9 (9.5)
**Tumor type**	
Lung cancer (NSCLC: *n* = 20, small cell lung cancer: *n* = 5)	25 (26.3)
Esophageal carcinoma	17 (17.9)
Liver cancer	11 (11.6)
Head and neck cancer	8 (8.4)
Genital system cancer	6 (6.3)
Colorectal cancer	7 (7.4)
Gastric carcinoma	7 (7.4)
Urogenital carcinoma	4 (4.2)
Cutaneous soft tissue carcinoma	3 (3.2)
Melanoma	2 (2.1)
Gallbladder carcinoma and bile duct carcinoma	2 (2.1)
Others	3 (3.2)
**TNM clinical classification**	
III	29 (30.5)
IV	58 (61.1)
Unknown	7 (7.4)
**Treatment lines at initiation of anti-PD-1/PD-L1 therapy**	
First-line	36 (37.9)
Second-line	29 (30.5)
Third-line and above	30 (31.6)
**Treatment**	
Immunotherapy	38 (40)
Immunotherapy + targeted therapy	22 (23.2)
Immunotherapy + chemotherapy	31 (32.6)
Immunotherapy + chemotherapy + targeted therapy	4 (4.2)
**Baseline ACE**	
Mean ± SD	0.12 ± 0.017
**Baseline PLR**	
Mean ± SD	204.899 ± 102.712
**Baseline NLR**	
Median	3.381
Range	1.021–40.625

The incidence of irAEs was 55.8%. Rash, immune-associated pneumonia, and hepatotoxicity were present in eight, seven, and 11 patients, respectively (Table 2).

**Table 2:** Description of irAEs occurring in individual patients

irAEs category	Number of patients with irAEs (%)
**Cutaneous**	
Rash	8 (8.4%)
Pruritus	2 (2.1%)
Vitiligo	1 (1.0%)
**Reactive cutaneous capillary endothelial proliferation**	5 (5.2%)
**Endocrine-related events**	
Hypothyroidism	3 (3.1%)
Diabetes	1 (1.0%)
**Hepatotoxicity**	
ALT/AST elevation	11 (11.6%)
**Gastrointestinal toxicity**	
Diarrhea	3 (3.1%)
Gastrointestinal bleeding	1 (1.0%)
**Immune-associated pneumonia**	7 (7.3%)
**Cardiac toxicity**	5 (5.2%)
**Hematological toxicity**	
Leukopenia	4 (4.2%)
Thrombocytopenia	4 (4.2%)
Anemia	7 (7.3%)
**Others**	
Increased creatinine	2 (2.1%)
Peripheral neuropathy	2 (2.1%)
Shingles	1 (1.0%)
Thromboembolism	1 (1.0%)
Hippocampal inflammation	1 (1.0%)
Fatigue	3 (3.1%)
Amylase and lipase elevation	1 (1.0%)
Oral mucositis	1 (1.0%)
**Total patients irAEs**	53 (55.8%)

The mechanism of cardiotoxicity associated with ICIs is not yet fully understood. Cardiotoxicity here mainly includes myocarditis, heart failure, or myocardial infarction.

*Abbreviation*: *irAEs* immune-related adverse events

### Associations between irAEs and anti-PD-1/PD-L1 inhibitor responses

ORR in the irAEs and no-irAEs groups were 13.2% and 11.9%, respectively; corresponding DCRs were 60.4% and 69.0%. There were no significant differences in ORR and DCR between the two groups (*P* = 0.763 and *P* = 0.381; Table 3).

**Table 3:** ORRs and DCRs of irAEs and no-irAEs groups

irAEs (*n* = 53)		no-irAEs (*n* = 42)		*P* value
*n*	%	*n*	%	
7	13.2	5	11.9	0.763^a^
32	60.4	29	69.0	0.381^b^

*Abbreviations*: *irAEs* Immune-related adverse events, *ORR* Objective response rate, *DCR* Disease control rate

^a^Continuity correction; ^b^Pearson chi-squared

**Fig. 1:**
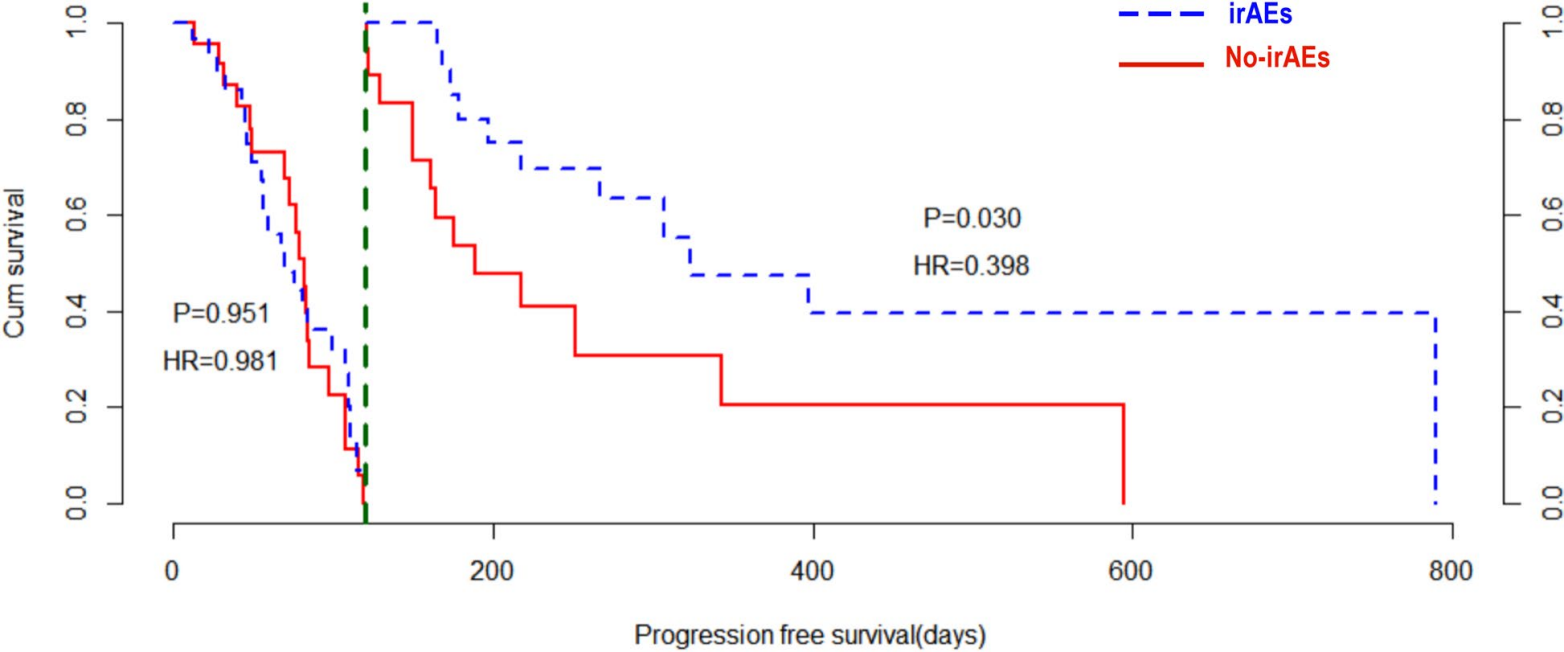
Landmark analysis according to the presence of irAEs. Kaplan–Meier curves with the threshold of 120 days (landmark analysis) for progression-free survival. Abbreviations: irAEs, immune-related adverse events; HR, hazard ratio

Considering the immortal time bias of irAEs, PFS was studied using landmark analysis (Fig. 1). Using 120 days as a threshold, the survival data were divided into two sections for survival analysis and a Kaplan–Meier curve was generated. The risk of disease progression was 0.981-fold in the irAEs group, compared with the risk in the no-irAEs group; there was no significant difference in PFS between the two groups (*P* = 0.951). After 120 days, the risk of disease progression was 0.398-fold in the irAEs group, compared with the risk in the no-irAEs group; the PFS was better in the irAEs group than in the no-irAEs group (*P* = 0.030).

### Peripheral blood predictive markers for irAEs

Using irAEs as the result variable, we generated ROC curves of NLR, PLR, and AEC; the respective cutoff values selected were 8.58, 180.68, and 0.045 × 10^9^/L. Based on cutoff value grouping, we compared the incidences of irAEs among groups; the incidences of irAEs were higher in the low NLR group (59.3%) than in the high NLR group (22.2%; *P* = 0.041; Table 3). In addition, the incidence of irAEs was significantly higher in the high-AEC group (63.0%) than in the low-AEC group (31.8%; *P* = 0.010; Table 4).

**Table 4:** Associations between peripheral blood markers and irAEs

Blood parameter	Cutoff value	irAEs, *n* (%)	*P* value
NLR	8.58		0.041*
Low (*n* = 86)		51/86 (59.3%)	
High (*n* = 9)		2/9 (22.2%)	
PLR	180.68		0.089
Low (*n* = 50)		32/50 (64%)	
High (*n* = 45)		21/45 (46.7%)	
AEC	0.045 × 10^9^/L		0.010*
Low (*n* = 22)		7/22 (31.8%)	
High (*n* = 73)		46/73 (63%)	

*Abbreviations*: *NLR* Neutrophil-to-lymphocyte ratio, *PLR* Platelet-to-lymphocyte ratio, *AEC* Absolute eosinophil count

^*^P < 0.05.

### Univariate and multivariate logistic analysis of predictive markers for irAEs

The results of univariate and multivariate logistic analyses are shown in Table 5. In univariate logistic analysis, good ECOG score (0–1), low-NLR (cutoff value: 8.58), and high-AEC (cutoff value: 0.045 × 10^9^/L) were important predictors of irAEs (*P* = 0.0499, OR: 0.196, 95% CI 0.038–1.000; *P* = 0.0499, OR 0.507, 95% CI 0.241–1.065; *P* = 0.012, OR: 3.651, 95% CI 1.322–10.076). Multivariate logistic analysis was performed involving factors with *P* < 0.2 in univariate analysis and tumor species; high-AEC and good ECOG score were independently associated with irAEs (*P* = 0.014, OR 4.114, 95% CI 1.328–12.858; *P* = 0.046, OR 0.159, 95% CI 0.026–0.970). In addition, immunotherapy combined with targeted therapy was associated with a greater risk of irAEs, compared with other treatments (*P* = 0.005, OR 0.156, 95% CI 0.045–0.544).

**Table 5:** Univariate and multivariate logistic regression analyses of irAEs

	Univariate analyses			Multivariate analyses		
	*P* value	OR	95% CI	*P* value	OR	95% CI
**Sex**	0.330	0.646	0.268–1.555	—	—	—
**Age**	0.975	1.001	0.957–1.046	—	—	—
**ECOG**	0.0499*	0.507	0.241–1.065	0.046*	0.159	0.026–0.970
0–1 ^c^						
2						
**Tumor type**	0.795	1.018	0.892–1.161	0.770	—	—
**TNM clinical classification**	0.508	0.786	0.385–1.605	—	—	—
**Treatment line**	0.150	1.939	0.787–4.777	0.69	2.908	0.922–9.169
First-line and second-line ^c^						
Third-line and above						
**Treatment**	0.125	0.780	0.567–1.072	0.044*	—	—
Immunotherapy ^c^				Base	—	—
Immunotherapy + targeted therapy				0.005*	0.156	0.045–0.544
Immunotherapy + chemotherapy				0.301	0.550	0.178–1.706
Immunotherapy + chemotherapy + targeted therapy				0.383	0.363	0.037–3.533
**NLR**	0.0499*	0.196	0.038–1.000	0.505	0.501	0.066–3.816
Low (≤ 8.58) ^c^						
High (> 8.58)						
**PLR**	0.091	0.492	0.216–1.120	0.216	0.537	0.200–1.440
Low (< 180.68) ^c^						
High (≥ 180.68)						
**AEC**	0.012*	3.651	1.322–10.076	0.014*	4.114	1.32–12.858
Low (≤ 0.045 × 10^9^/L) ^c^						
High (> 0.045 × 10^9^/L)						

*Abbreviations*: *ECOG* Eastern Cooperative Oncology Group, *NLR* Neutrophil-to-lymphocyte ratio, *PLR* Platelet-to-lymphocyte ratio, *AEC* Absolute eosinophil count, *OR* Odds ratio, *CI* Confidence interval

^*^P < 0.05. ^c^Comparison reference in multivariate analyses

### Forest plot of multivariate logistic regression analyses for irAEs

**Fig. 2:**
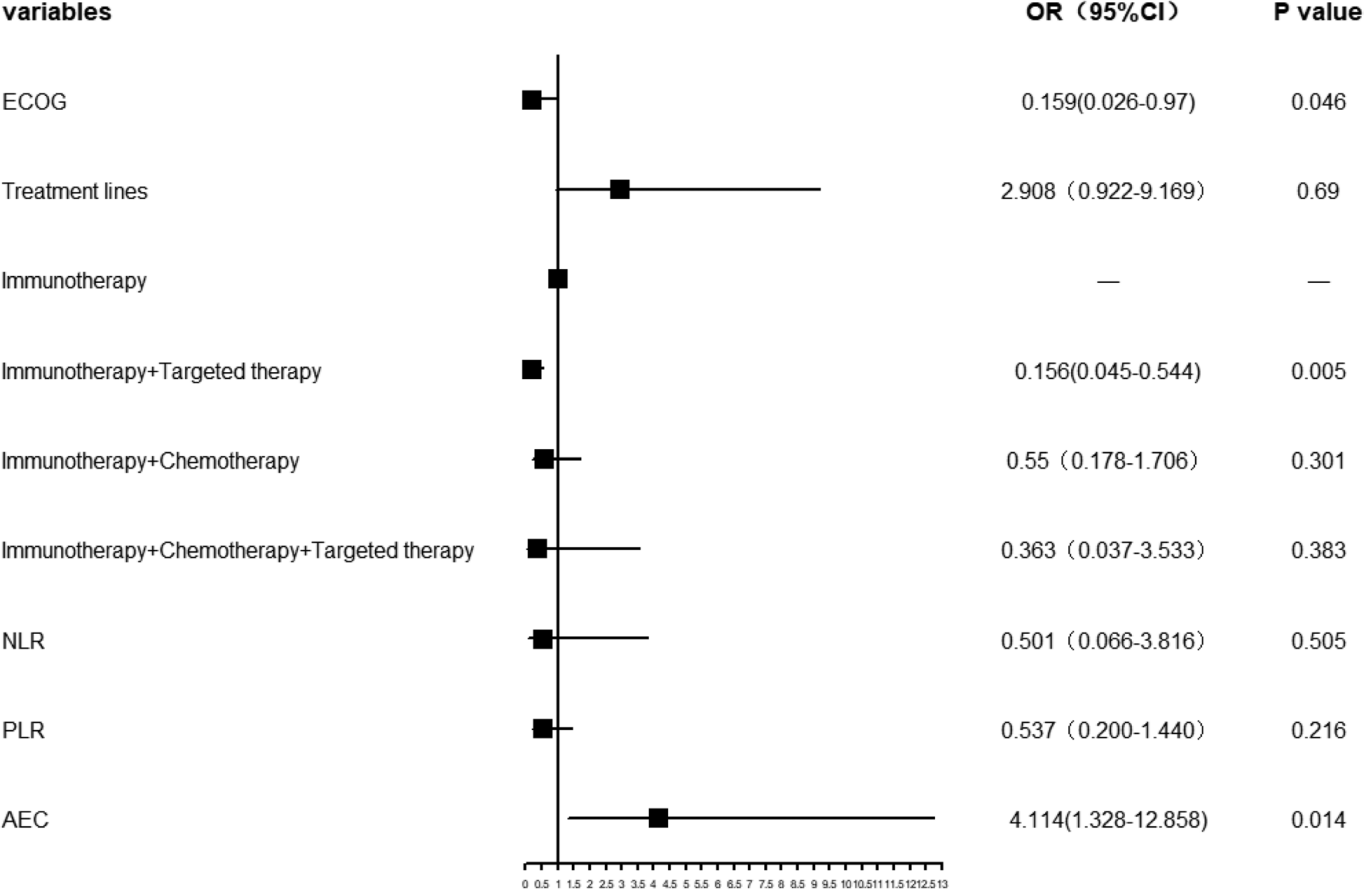
Forest plot of multivariate logistic regression analyses for irAEs. The vertical line in the middle of the figure is the invalid line (i.e., OR: = 1). Each horizontal line is the line between the upper and lower limits of 95% CI of the study; the length of each line segment intuitively represents the size of 95% CI. The small square in the center of the horizontal segment is the position of the OR value; its size reflects the weight of the study. The reference of comparative subgroups was the same as the multivariate logistic analysis of predictive markers for irAEs. Abbreviations: ECOG, Eastern Cooperative Oncology Group; NLR, neutrophil-lymphocyte ratio; PLR, platelet-lymphocyte ratio; AEC, absolute eosinophil count; OR, odds ratio; CI, confidence interval

To more intuitively depict the results of multivariate logistic analysis of irAEs, we generated a forest plot with irAEs as the study event (Fig. 2). The incidence of irAEs was higher in the high-AEC group than in the low-AEC group; the incidence of irAEs was greater in the good ECOG PS (0–1) group than in the ECOG PS (2) group. Similarly, the incidence of irAEs was lower in patients receiving immunotherapy combined with targeted therapy, compared with patients receiving other treatments.

### Associations of baseline AEC with anti-PD-1/PD-L1 efficacy

**Fig. 3:**
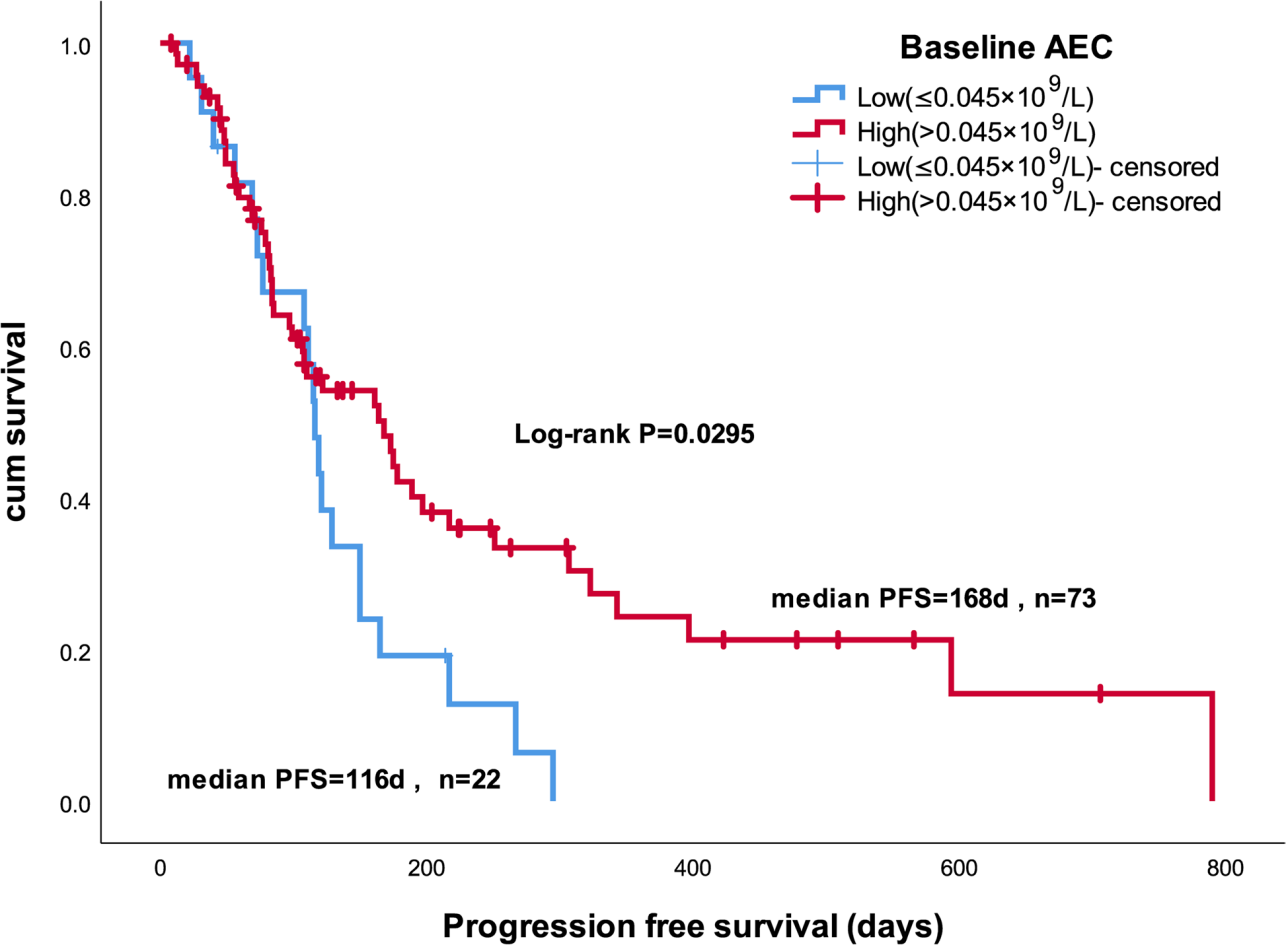
Kaplan–Meier survival curves for progression-free survival stratified according to baseline AEC cutoff, determined by ROC curve analysis

The ORRs of the high-AEC (> 0.045 × 10^9^/L) and low-AEC (≤ 0.045 × 10^9^/L) groups were 9.5% and 22.7%, respectively; the corresponding DCRs were 64.3% and 63.6%. There were no significant differences in ORR (*P* = 0.208) or DCR (*P* = 0.949) between the two groups (Table 6). The median PFSs in the high-AEC (> 0.045 × 10^9^/L) and low-AEC (≤ 0.045 × 10^9^/L) groups were 168 and 116 days, respectively (*P* = 0.0295; Fig. 3).

**Table 6:** ORRs and DCRs of high-AEC and low-AEC groups

	High-AEC (*n* = 73)		Low-AEC (*n* = 22)		*P* value
	*n*	%	*n*	%	
ORR	7	9.5	5	22.7	0.208^a^
DCR	47	64.3	14	63.6	0.949^b^

*Abbreviations*: *AEC* Absolute monocyte count, *ORR* Objective response rate, *DCR* Disease control rate

^a^Continuity correction, ^b^Pearson chi-squared

## Discussion

Immune checkpoint inhibitors such as anti-PD-1/PD-L1 inhibitors have become crucial therapeutic options for patients with advanced malignant tumors, but the associated irAEs may lead to treatment interruption or fatal disease [10–12]. Early prediction and correct treatment are critical for irAE management.

pD-L1 expression level, smoking history, and other factors have demonstrated close relationships with malignant tumor prognosis in patients receiving immune checkpoint inhibitors and other treatments [41–45]. The relationships between irAEs and anti-PD-1/PD-L1 inhibitor responses in advanced malignant tumors have long been controversial. A recent meta-analysis of 30 studies showed that irAEs (especially endocrine, cutaneous, and low-grade irAEs) were significantly associated with PFS and overall survival in patients with advanced malignant tumors who were receiving anti-PD-1/PD-L1 inhibitors; however, that meta-analysis did not examine ORR [46]. Another study showed that combined immunotherapy was effective, but the incidence of serious adverse events (grade 3 or higher) was lower [47]. In the present study, we found no significant differences in ORRs and DCRs between irAEs and no-irAEs groups, similar to previous results [37, 38], although an association between irAEs and PFS was not directly observed. Because of the immortal time bias of irAEs and the intersection points in the overall analysis, we used landmark analysis, in which the irAEs group showed a survival advantage after PFS for 120 days. This finding is related to the initial onset of irAEs: most irAEs reportedly appear within 3 months after the initiation of treatment, while serious adverse reactions such as immune-associated pneumonia appear within 2 months [48]. Our clinical data indicated that some patients discontinued treatment early because of severe adverse reactions such as immune-related myocardial injury and immune-related pneumonitis.

Peripheral blood markers such as baseline NLR and PLR have shown predictive value in the efficacy of anti-PD-1/PD-L1 inhibitors in advanced malignant tumors [49–54], as well as in the possibility of predicting the risk of irAE occurrence [15–17]. Moreover, the eosinophil count is increased in malignant tumors [55, 56], and the presence of eosinophils in the peripheral blood has been associated with irAEs [17, 18, 30]. Changes in lymphocyte percentages, as well as the counts of neutrophils, eosinophils, and mononuclear cells, are presumed to reflect baseline immune function [57]. In the present study, we assessed the predictive values of baseline NLR, PLR, and eosinophils for the risk of irAEs; we found that the incidences of irAEs were significantly higher in the baseline low-NLR and baseline high-AEC groups than in the high-NLR and low-AEC groups. Previous studies showed that higher baseline NLR predicted poor efficacy of anti-PD-1/PD-L1 inhibitors, indirectly suggesting the potential for a positive correlation between irAEs and treatment efficacy. Furthermore, univariate logistic analysis showed that both baseline low-NLR and baseline high-AEC were risk factors for irAEs; following correction for confounding factors (e.g., tumor type, treatment, and treatment line) multivariate logistic analysis showed that only AEC was an independent factor associated with irAEs. Baseline PLR reportedly can be used as an independent predictor of irAEs in the immune checkpoint inhibitor treatment of patients with advanced non-small cell lung cancer [15]; however, our multivariate analysis results did not support the above conclusion. We speculate that baseline AEC may have greater value in the prediction of irAE occurrence, compared with baseline NLR and PLR. To our knowledge, this is the first comparison of the predictive values of baseline NLR, baseline PLR, and baseline AEC for irAEs.

Accordingly, we investigated the relationship between baseline AEC and anti-PD-1/PD-L1 efficacy. We found no difference between high-AEC and low-AEC in terms of ORR, in contrast to the results of previous studies. However, we found that PFS was significantly better in the high-AEC group than in the low-AEC group [30].

Notably, we found that irAEs were more likely to occur in patients with good ECOG PS, similar to previous findings [35]. Following adjustment for confounding factors (e.g., tumor type, treatment, and treatment line), we found that good ECOG remained an independent positive predictor of irAEs. In addition, treatment lines are reportedly related to irAEs, such that second-line treatment and above is more likely to lead to irAEs [35], in contrast to our findings. In several studies, combination treatment comprising immunotherapy plus sequential or concurrent treatment with cytotoxic chemotherapy has been reported to increase the risk of irAEs [58]. Notably, we found that the incidence of irAEs was low in patients receiving immunotherapy combined with targeted therapy; because there are no other published data regarding irAEs in this patient population, additional large prospective studies of single tumor species are needed to confirm our findings.

There were a few limitations in this study. First, this was a single-center retrospective study. Second, this study may have underestimated the influences of hormones or immunosuppressants on irAE classification. Finally, because of the retrospective study design, we did not investigate some factors, such as infection, allergy, and drug use; we thus ignored the potential influences of these factors on the results. Therefore, multi-center, prospective studies are needed to validate our results.

## Conclusion

Our findings indicate that baseline AEC and ECOG PS can be used as independent predictors of irAE occurrence to guide clinical practice, provide early warning of irAEs, and ensure preventive measures against irAE onset, thus aiding in the correct management of irAEs.

## Data Availability

The datasets generated and/or analyzed during the current study are not publicly available due to personal privacy but are available from the corresponding author on reasonable request.

## Abbreviations

ECOG: Eastern Cooperative Oncology Group
NLR: Neutrophil-to-lymphocyte ratio
PLR: Platelet-to-lymphocyte ratio
AEC: Absolute eosinophil count
HR: Odds ratio
CI: Confidence interval
irAEs: Immune-related adverse events
ORR: Objective response rate
DCR: Disease control rate
ICIs: Immune checkpoint inhibitor
CR: Complete response
PR: Partial response
SD: Stable disease
PD: Progressive disease
PFS: Progression-free survival
ROC: Receiver operating characteristic
ECOG: Eastern Cooperative Oncology Group
PS: Performance status
